# On the Curative Medication of Intermittent Fever

**Published:** 1846-08

**Authors:** 


### PART VI.—SELECTIONS.

1. *On the Curative Medication of Intermittent Fever.* By M. Bretonneau.—A sort of drunkenness, more or less painful, produced by a single and suitable dose of sulphate of quinine, repeated, if necessary, two days afterwards, suppresses for eight days *simple* intermittent fever.

In the same way, that a person who is in the habit of intoxication, we find that much wine may be taken without producing this condition; so we every day see individuals affected with ague taking great quantities of quinine, without the fever becoming suppressed, or its return being prevented.

M. Bretonneau agrees with the Jesuits who first imported cinchona, and learned how to administer it; and with Torti, who for thirty years practised in the hospital at Tours, that every sufficient dose of bark loses its febrifuge power by fractioning it, exactly as a dose of wine loses its intoxicating property by being divided.

It has been stated that the prolonged administration of many doses, whose sum altogether amounted to several efficient doses, has completely failed. M. Bretonneau has seen a quartan fever, which had resisted two ounces of bark, yield to two drachms of the same medicine; but the two drachms were given at once, while the two ounces were taken in very small quantities at a time, and during fifteen days.

Small doses, which habituate a patient to the action of cinchona, injure the beneficial results of adequate doses; they hurt the digestive apparatus, and render the febrifuge intoxication more difficult to obtain.

M. Bretonneau corroborates the statements of Sydenham and Morton—that the dose of bark which has suppressed a paroxysm, if repeated before the supposed epoch of its return will prevent it; and, moreover, the immunity thus procured may be prolonged by renewed doses exhibited at gradually lengthened intervals, until a perfect immunity from relapse becomes established. The progression recommended in the giving of these preservative doses is the following:—A second dose equal to that which suppressed the fever is to be exhibited, according to the nature of the fever, on any day from the first to the sixth interval, then to be repeated after intervals of 7, 8, 9, 10, 12, 14, 16, 18, 22, 30 days. The best time for giving each preservative dose is immediately after a light dinner; and the first dose had better be given shortly after the decline of the suppressed access, so as to be as distant as possible from the next threatened paroxysm. A relapse will render it necessary to renew the treatment from the commencement. The preservative doses should be approximated if it is found that the accesses become more frequent.

Unusual exercise, the prolonged impression of cold, indigestion, purging, &c., provoke the return of the fever which ordinarily, with the precautions indicated, does not return till spring. Occasionally, at Tours, the fever which had been suppressed, but its relapse not guarded against by the preservative treatment mentioned above, was accustomed to become renewed during twenty or thirty months.

M. Bretonneau has observed that an adequate dose of sulphate of quinine commonly produces vertigo and noises in the ears; subsequently, at a period more or less elongated from the first effect, there frequently supervenes a febrile state, which there is danger of confounding with a return of the intermittent fever. This sort of fever is of good augury. During its continuance the skin is hot, the pulse elevated, and this state corresponds to that condition of febrile reaction, which it is so very erroneous as to dread in paludial affections. With extreme sagacity, Dr. Bally discovered, in 1821, that in Paris, agues became developed in the spring, in subjects who had spent the autumn in localities where the fever prevailed. Evidence, the most irrefragable, bore out this assertion, nothing similar having been observed in persons who had not left Paris. M. Bretonneau has found, that a fever developed under these circumstance has all the tenacity prevalent in the district where it was contracted.

From fifteen to twenty grains of the sulphate of quinine, or from three or four drachms of good cinchona, suffice to suppress the fever of an adult, and to keep it suppressed during eight or nine days. Many reasons lead to the belief, that it is useful that the necessary dose should not be exceeded.

Intermittent fever is endemic in the locality where these observations have been a multitude of times repeated; and the number of fever patients admitted into the hospital has been so great, that in this establishment, previously to the discovery of quinine, 1200 lbs. of cinchona have been prescribed in the course of ten years, and since that discovery, frequently 50 oz. of sulphate of quinine have been prescribed in the pharmacy during the three months of autumn.

If a serious and unusual symptom shows itself for the second time in the course of a fever, whose paroxysms have been but little marked; if a lethargic torpor, faintings, alvine evacuations, either bilious, or like the washings of flesh, or melanic; if a severe epigastric pain, a superabundant sweat, a marble coldness, shiverings, symptoms exceeding an ordinary severity, accompanied by prostration, nearly complete abolition of the pulse; if these symptoms become developed, and prolong the paroxysm beyond the previous term of its duration, the dose should be doubled and given before the complete decline of the paroxysm—the fever has now become *pernicious.* It is necessary to guard this large dose with a third of a grain of watery extract of opium, or four or five drops of wine of opium. If tolerance on the part of the stomach can be obtained, it will be necessary to inject the febrifuge into the rectum. The intestinal rejection is most easily retained if it produces neither a sensation of heat nor cold, if it is deposited above the second sphincter—a region less excitable than that which is below it, if it is small in bulk, (from three to four ounces) and if made to traverse a large pipe slowly; a mixture of two drachms of powdered cinchona and a scruple of sulphate of quinine, is more easily retained than a solution of half a drachm of sulphate of quinine.

A moderately abundant and substantial diet aids powerfully the good success of preservative treatment. This is what Sydenham and Morton, one hundred and fifty years ago expressly affirmed.—*Dublin Hospital Gaz.*, Dec. 1, 1845, from *Jour. de Méd.*, par Trousseau, March, 1846, in *Amer. Jour. of Med. Sciences.*

